# Multi-Sensors for Human Activity Recognition

**DOI:** 10.3390/s23104617

**Published:** 2023-05-10

**Authors:** Athina Tsanousa, Georgios Meditskos, Stefanos Vrochidis, Ioannis Kompatsiaris

**Affiliations:** 1 Centre for Research and Technology Hellas, Information Technologies Institute, 6th Km Charilaou-Thermi, 57001 Thessaloniki, Greece; stefanos@iti.gr (S.V.); ikom@iti.gr (I.K.); 2 School of Informatics, Aristotle University of Thessaloniki, 54124 Thessaloniki, Greece; gmeditsk@csd.auth.gr

## 1. Introduction

Human activity recognition (HAR) has made significant progress in recent years, with growing applications in various domains, and the emergence of wearable and ambient sensors has provided new opportunities in the field. The use of multi-sensor systems, which integrate data from multiple sensors, has the potential to improve the accuracy and reliability of human activity recognition. This Special Issue of *Sensors* on “Multi-Sensors for Human Activity Recognition” brings together works from various domains to share their findings on a variety of aspects regarding the monitoring of human activity. This Special Issue focused on the current state-of-the-art works related to the broader field of HAR, with a special emphasis on multi-sensor environments. The following section offers a summary of the nine featured articles regarding applications of HAR in smart homes, speech and gesture recognition and security issues of IoT systems, among others.

## 2. Overview of Contribution

Activity recognition can have applications in the security domain, for surveillance purposes, which is usually achieved through visual sensors. The types of activities relevant to this application are violent actions. There are already available databases regarding general violence detection; however, the authors of [1] contributed to this field with a large set of annotated data, the Bus Violence dataset, with violent actions entirely located on public transport. The dataset is a large collection of video clips from multiple cameras for detecting simulated acts of violence on public transport. The paper also presents an application of DL (deep learning) methods for detecting harmful activities. 

Assisted living environments are probably the field with the most applications of HAR. Numerous works can be found in the relevant literature, testing a variety of methods and sensors for monitoring the activities of people living in these environments and for detecting harmful events. This paper [2] tested the performance of algorithms for multi-resident activity recognition, which is approached as a multilabel classification (MLC) problem. Using two public datasets (ARAS and CASAS), the authors tested the following algorithms: the random k-labelsets algorithm (RAkELd), an ensemble method for multilabel classification; binary relevance; and the classifier chain method, which is another widely used MLC ensemble method and constructs a chain of binary classifiers, where the number of classifiers is the same as the number of labels in the dataset. 

This review [3] presents the challenges of the applications of HAR in smart homes, the algorithms and works in this field and any identified gaps. The authors divide the HAR systems into two categories: video-based and sensor-based systems. Video-based systems raise some privacy issues. The authors review papers including data-driven approaches (DDA) and knowledge-driven approaches (KDA) for HAR. There is a detailed reference to methods for feature extraction, an important step in sensor data modeling. The segmentation of temporal data is also discussed. 

In the sports field, the interest of researchers lies in a subfield of HAR, pose estimation. Its applications can be found in everyday apps in smartwatches and smartphones for monitoring exercise, burnt calories, etc. HAR systems can be adapted to be applied in specific sports and detect certain movements. Likewise, in [4], the authors employed computer vision in the martial arts domain, aiming to identify postures performed by karatekas. The authors proposed a system that will be able to recognize the correct execution of an entire series of movements by a karateka. 

An important aspect of multi-sensor systems, besides their performance in recognition tasks, is the secure exchange and storage of data. The authors in [5] explored the security challenges of an IoT localization system and proposed a blockchain-based distributed paradigm to secure localization services. The proposed system is strongly focused on the protection of the users’ privacy.

A sub-category of HAR is the recognition of hand gestures that can be exploited to control home appliances and robots or to assist in communication with people who are deaf and people who cannot speak. In [6], a system with dual cameras was proposed for both static and dynamic gesture recognition. The authors propose a hardware architecture that improves execution speed while maintaining high efficiency.

In the broader context of monitoring human activity, this Special Issue published a paper [7] monitoring the use of facial masks in the COVID-19 era and specifically the exhalation of CO_2_ from people wearing four different types of masks. Using a multi-sensor system of four low-cost carbon dioxide (CO_2_) sensors, the authors measured CO_2_ in two indoor spaces and created spatial heatmaps to visualize the CO_2_ concentration. 

Besides data-driven approaches, knowledge-based approaches are also adopted in multi-sensor IoT environments to allow interoperability and represent data and events. The authors in [8] presented a semantic web approach for detecting lifestyle and health-related activities from wearable sensors, in a use case about improving the care of patients with multiple sclerosis (MS). The paper described a lightweight framework for detecting lifestyle and health-related problems and supporting the integration of a variety of lifestyle wearable sensors. To achieve interoperability at different levels, the authors used OWL 2 ontologies to generate interoperable knowledge graphs that are aligned with existing vocabularies and conceptual models.

An application of HAR regarding safety while driving is presented in [9], where the authors propose a system for voice and hand gesture recognition, which monitors human-vehicle interaction and replaces the driver’s need to control the in-vehicle infotainment system, reducing distractions for the driver and, consequently, possible fatal accidents. The authors applied sensor fusion techniques to perform multi-sensor monitoring and a binarized convolutional neural network algorithm to reduce the computational workload of the CNN in classifying speech and hand commands. 
